# Youth sports activity and young people’s well-being after a disaster: a trial with the Mastery Approach to Coaching (MAC) in the Philippines

**DOI:** 10.1186/s13104-018-3860-1

**Published:** 2018-10-22

**Authors:** Takeshi Akiyama, Ernesto R. Gregorio, Jun Kobayashi

**Affiliations:** 1 Nagano College of Nursing, 1694 Akaho, Komagane, Japan; 2 Department of Health Promotion and Education, College of Public Health, University of the Philippines Manila/SEAMEO Regional Center for Public Health, Hospital Administration, Environmental and Occupational Health, Pedro Gil St., Ermita, Manila, Philippines; 3 Department of Global Health, School of Health Sciences, Faculty of Medicine, University of the Ryukyus, 207 Uehara, Nishihara, Okinawa Japan

**Keywords:** Mastery Approach to Coaching, Self-esteem, Adolescent, Sports activity, Post disaster, Philippines

## Abstract

**Objective:**

Sports activities is broadly utilized to support well-being of youth after a disaster or conflict. However, scientific validation of programs have not been conducted. The Mastery Approach to Coaching (MAC) is a coaching-education program on sports activities. The MAC reported to have a positive effect on youngsters’ self-esteem. As self-esteem is generally known to be beneficial for mental status, we tested the effect of a MAC program on students’ self-esteem in a disaster-affected area: Leyte, Philippines. We recruited 10th grade students from three schools; one school was allocated to the MAC intervention and the two schools to the control group. All schools were encouraged to involve students in volleyball from January to February 2015. In January 2015, MAC workshop was conducted in the intervention school before the sports activity.

**Results:**

A total of 293 students completed the questionnaires. The intervention school (n = 51) showed a significant change in self-esteem, with the mean score increasing from 20.2 to 21.1 (p = 0.02). Neither school in the control group showed the significant change. The result showed the feasibility and a positive effect of sports activity with the MAC. However, further investigation should be conducted.

*Trial registration* UMIN Clinical Trials Registry ID: UMIN000033197 on June 30th 2018. Retrospectively registered

## Introduction

Sports and recreational activities had been suggested and implemented as a psychosocial support to children and adolescents after a catastrophic event, such as disaster and armed conflict [1–5]. International organizations and humanitarian aid agencies had been implementing sports programs in the affected areas worldwide [3]. Although the term sports can be used interchangeably with physical activity and exercise, sports activity can be defined as those which “involve physical activity and exercise but differ in that they also have a set of rules, or goals to train and excel in specific athletic skills [7].”

Sports activities are intrinsic and naturally occurring and can have a stabilizing impact on individuals and communities even after a disaster through human relationships and connectedness [4, 6], with sustaining and enhancing children’s existing resilience factors and trust in a positive future [4]. However, little is known about how sports programs are effective or might be useful in addressing mental health problems in children [1, 4, 6]. Few programs were designed scientifically and quantitatively verified the effect of sports activity on the well-being of children and adolescents in an affected area.

Self-esteem is known to have a protective role against mental health problems such as stress, and depression [8]. Although the mechanism and longitudinal effect are still being studied, studies suggest a beneficial effect of sports activity on self-esteem of the youngsters [9–11]. The Mastery Approach to Coaching (MAC) is a coaching-education program that has been reported to have a positive effect on young people’s personal and social development through sports activities [12, 13]. In particular, the MAC has been reported to have a significant effect on the improvement of young people’s self-esteem [14, 15]. The MAC was derived from the framework of goal orientation theory [12, 13]. Besides the MAC, there are few validated coaching-education programs on personal, social, and athletic development of children and adolescents [12, 13].

The MAC approach instructs how to create an educational environment; the mastery motivational climate where commitment to making progress are emphasized and minimizing pressure [16]. In a study that provided an intervention for the self-esteem of 62 boys (mean age = 11.39, SD = 0.81 years), a significant improvement was found among those who had lower self-esteem before the intervention [15]. The MAC stands out because of its feasibility as verified by a number of studies in the field [17]. Furthermore, the MAC has developed a manual and DVD for the general public [13] and it could be applicable in broad school settings.

In the Philippines, typhoon Haiyan, locally known as “Yolanda,” caused over 6000 deaths and a displacement of more than 4 million people in November 2013. The Philippines is a developing country with limited resources for mental health services. In order to test the feasibility and verify the potential of the sports activity with MAC, we conducted a trial of the MAC on the students’ self-esteem in Leyte, Philippines.

## Main text

### Methods

This is a quasi-experimental trial in Leyte, Philippines. The study was implemented from January to February 2015, approximately a year after the onslaught of Typhoon Haiyan in November 2013. We targeted 10th grade students being the highest level in the junior high school. Of 109 public schools which had 10th grade in Leyte, we included three (Schools A, B, C). We targeted all 10th grade students (n = 404) of those three schools. One school (School A) was assigned to the MAC-based intervention group, and the other two schools (Schools B, C) were included in the control group.

All participating schools were encouraged to involve all students. Volleyball was chosen based on the feedback from the students and teachers. In all the schools, one male and one female team were formed per class. The researchers recommended that the schools hold the sports activity at least 1 h per day for 4 days a week. However, the schools could include the activity during their extracurricular or formal physical education activities. The duration of the activity was 2 months, from January to February 2015. The final goal of the activity was to set intra-school tournaments among the male and female teams. The number of days engaged in sports activity was counted by the teachers. Students’ shoes, balls, net, and poles were provided to all the schools.

In January 2015, a MAC workshop was conducted in School A before the sports activity began. The researchers targeted and invited student leaders [selected by the school; two males and two females per class (section)] and teachers to serve as coaches for each class. A total of 20 students and 8 teachers participated in the MAC and in the actual coaching sessions. The translated MAC manual was delivered to the participants, and a DVD with instructions and advice from the researchers were provided.

Students’ self-esteem was measured using the Rosenberg’s Scale which was translated into the local language. However, indices of Cronbach’s alpha were poor: 0.50 in the pre-intervention and 0.51 in the post-intervention. Back translation was conducted to check the precision of the forward translation. A t-test was used to examine the change in the average scores on the questionnaire. Statistical significance was set at 0.05. The analysis was conducted using IBM SPSS Windows version 22.

### Results

Of the targeted 404 students, 364 participated in the activity. As a result, only 293 (72.5%) of 404 students completed both the pre- and post-intervention questionnaires. The mean age of these participants was 16.6 years (SD = 1.6) as shown in Table 1. The intervention group indicated as school A included 51 students. The control group (schools B and C) included 242 students.

**Table 1:** Characteristics of the participants (n = 293)

	*n*	(%)	Mean age (years)	SD
Total	293	(100.0)	16.6	1.61
Male	127	(43.3)	16.9	1.53
Female	166	(56.7)	16.3	1.63
Intervention (n = 51)				
School A	51	(17.4)	16.4	1.08
Control (n = 242)				
School B	21	(7.2)	16.2	1.12
School C	221	(75.4)	16.6	1.74

In the pre-intervention evaluation, the intervention and control groups did not show a significant difference in the mean score of self-esteem (Table 2). However, there was a significant difference in the mean number of engaged days between the intervention and control groups. Furthermore, the self-esteem score on the post-intervention evaluation was higher in the intervention group than in the control group (p < 0.05). The intervention group showed a significant change in the self-esteem, with the average score increasing from 20.2 to 21.1 (p = 0.02).

**Table 2:** Scores on the Rosenberg’s self-esteem scale in pre-and post-intervention

	Pre		Engaged days		Post		p value^a^
	Mean	SD	Mean^b^	SD	Mean^b^	SD	
Intervention							
School A (n = 51)	20.2	2.36	23.4	10.30	21.1	2.81	0.02
Control (n = 242)	19.7	3.05	18.6	2.92	19.7	3.28	0.83
School B (n = 21)	19.2	2.86	18.1	2.56	20.2	3.26	
School C (n = 221)	19.7	3.07	18.6	2.97	19.7	3.26	

^a^The t-test results on the difference between pre and post intervention

^b^The difference between the intervention and control groups was statistically significant (p < 0.05)

### Discussion

The results suggest a big potential in using a sports-based activity with MAC in improving students’ self esteem after a disaster. Such an intervention could be an option to support the mental health needs of those in the affected areas. Although the target would be limited to those who participated in the sports activity, it could also be an option in population-based approaches. Furthermore, this study was conducted in a school setting which provide an evidence that schools could be an important resource in supporting the overall physical health and mental health of children and adolescents. Such school-based approach can optimize the resources already available in the school [18–20].

## Limitations

The above findings should be taken in the light of this study’s possible limitations. The first consideration is the research design of this study. The intervention consisted of only one school and a small number of participants in the control group; therefore, a larger sample size and randomization should be conducted. In addition, the reliability of the translated questionnaire was low as indicated by the indices of Cronbach’s α. Although we conducted back-translation with different translators, the translation process should have been examined more carefully and the unique variations in the local dialect could be considered. Moreover, using a simpler self-esteem scale might be an option for this issue [21].

The experimental group had more number of days of exposure to the intervention compared to the control and this might have affected the study result. It is recommended that the importance of adhering to the study protocol and the strict monitoring of attendance should be emphasized to the coaches in future studies. Overall, more validity and reliability in the methodology of the data collection and elaboration of the questionnaire are necessary in the future.

The improvement in the self-esteem scores of students in the intervention school was only 0.9; 20.2 at pre-intervention and 21.1 at post-intervention. Further research to validate the impact of the intervention on the well-being of students is needed.

We employed only a single sports activity. Different sports activities, such as individual sports [22] and differences in the skills of students might have affected the results [23]. Further investigation is necessary to examine students’ motivation, goal orientation, students’ characteristics, types of activities, self-efficacy and self-concept in sports, and social support [16, 23–26].

As mentioned above, the current study has a number of limitations. However, this is the first intervention study that looked into the effects of sports activity with MAC to students’ self-esteem. In a country where occurrences of disasters had become a new normal [27], such post-disaster activity would be meaningful and could be part and parcel of the school curricular and extra-curricular activities after a disaster. Moreover, these kinds of interventions timely respond to the mandate of the Republic Act No 11036 otherwise known as the Philippine Mental Health Act recently passed and signed into a law. This Act calls for the development of an age-appropriate strategy on mental health at all educational levels.

## Abbreviation

MAC: Mastery Approach to Coaching
